# Decellularized corneal-based 3D scaffolds: methods decellularization, characterization, mechanical properties, and species source

**DOI:** 10.1016/j.reth.2025.101044

**Published:** 2025-11-20

**Authors:** Vahid Akbaripour, Leila Rezaei, Gelavizh Rostaminasab, Farid Daneshgar, Omid Bahiraee, Leila Rezakhani

**Affiliations:** a Student Research Committee, Kermanshah University of Medical Sciences, Kermanshah, Iran; b Clinical Research Development Center, Imam Khomeini and Mohammad Kermanshahi and Farabi Hospitals, Kermanshah University of Medical Sciences, Kermanshah, Iran; c Fertility and Infertility Research Center, Health Technology Institute, Kermanshah University of Medical Sciences, Kermanshah, Iran; d Department of Tissue Engineering, School of Medicine, Kermanshah University of Medical Sciences, Kermanshah, Iran

**Keywords:** Decellularized cornea, Tissue engineering, Extracellular matrix (ECM), Recellularization

## Abstract

Corneal blindness is a significant worldwide health issue owing to low number of global corneal donors. Decellularized corneal scaffolds intend to be a promising choice for corneal repair by maintaining the native extracellular matrix (ECM) while minimizing immune reactions. This effort comprehensively reviews various decellularization strategies such as physical, chemical, and biological methods and their impact on ECM integrity, transparency, and mechanical strength. We explored source tissues such as porcine, human, and SMILE-derived lenticules based on their structure similarity and clinical suitability. Characterization techniques including immunohistochemical, histological, mechanical, and in vivo assessments are reviewed to evaluate scaffold quality and biocompatibility. Recellularization approaches which restore corneal functionality, using epithelial, stromal, and endothelial cells also have been investigated. Additionally, progresses in composite biomaterials and 3D bioprinting utilizing decellularized corneal matrices are highlighted, showing enhanced transparency, adhesion, and regenerative potential. Despite clinical progress which is evidenced by successful preclinical studies and clinical trials, some challenges such as protocols optimization, large-scale production, and integration with host tissue remain. Further integrated research is essential to optimize scaffold design, ensure long-term safety, and establish decellularized corneas as a possible solution to the scarcity of donor tissues for transplantation.

## Abbreviation

(ALDH1): Aldehyde dehydrogenase 1
(APCs): Acellular porcine corneas
(BACs): Bioartificial corneas
(CHAPS): 3-[(3-Chola-Midopropyl) Dimethylammonio]-1-Propanesulfonate
(DSMS): Decellularized squid mantle scaffold
(ECM): Extracellular matrix
(ECM): Ethylenediaminetetraacetic acid x
(GAG): Glycosaminoglycan
(αGal): Galα1-3Galβ1-4GlcNAc-R
(H&E): Hematoxylin and eosin
(HHP): High hydrostatic pressure
(LKP): Lamellar keratoplasty
(LSCD): Limbal stem cell deficiency
(NH4OH): Ammonium hydroxide
(PEG): Poly (ethylene glycol)
(PBS): Phosphate-buffered saline
(PLA2): Phospholipase A2
(scCO2): Supercritical carbon dioxide
(SLG): Sodium N-lauroyl glutamate
(SMILE): Small Incision Lenticule Extraction
(UHHP): Ultra-high hydrostatic pressure

## 1 Introduction

The cornea, the outermost protective covering of the eye, provides vital visual system functions such as optical refraction, visual transparency, and biomechanical protection. Additionally, the cornea can be easily damaged by external trauma and corneal infections, which in severe cases can result in blindness [1]. The Global Vision Database estimates that 36 million individuals are blind globally [2]. According to the World Health Organization, 5 % of all causes of blindness are caused by corneal opacities [3]. The only treatment available when corneal transparency is compromised is corneal transplantation [4]. Nonetheless, the global supply and demand for corneal tissues are out of balance [5]. The availability of high-quality donor tissue is arguably the largest obstacle to corneal transplantation at the moment [6]. This gap varies dramatically among regions, with westernized states generally well supplied [7,8], while demand in Africa and Asia far outstrips supply [8,9]. Tissue engineering has gained popularity as a potential solution to the corneal scarcity issue within the past 10 years. Complex characteristics necessary for corneal function should be present in tissue-engineered corneal scaffolds. Replicating a corneal scaffold from biomaterials is considerably more challenging due to these characteristics, which include (i) light transparency, (ii) resilience to ultraviolet light, (iii) insensitivity to photons, and (iv) specific tensile strength [10,11]. Building three-dimensional biomaterial scaffolds and seeding them with corneal cells has been the main focus of tissue engineering techniques. Theoretically, the cells will eventually produce new tissue by rebuilding the scaffold and excreting extracellular matrix (ECM) components. For this objective, a variety of polymeric biomaterials have been investigated [[12], [13], [14], [15], [16], [17]]. Nevertheless, creating scaffolds with structural and biochemical makeups comparable to the natural cornea has proven to be the biggest challenge to success. Although aligned nanofibers [18,19], collagen fibril magnet alignment [20] and plastic compression [21,22] are some techniques for adding structural characteristics that resemble the cornea to these scaffolds, they are still unable to completely replicate the intricate structure of the cornea. In order to create scaffolds that resemble the structure and composition of natural human cornea, decellularized corneal scaffolds have recently been proposed as an alternative to manufactured biomaterials. Both pre-clinical animal research and human clinical applications have effectively employed scaffolds made from decellularized tissues and organs [23]. Decellularization is the process of removing cellular and nuclear material from a tissue while leaving the ECM's functional and structural proteins intact [24]. The removal of cellular and immunogenic components from an organ or tissue while maintaining ECM with its natural micro- and macroscopic structure is made possible by the decellularization of a three-dimensional matrix scaffold, such as the cornea [25]. The methodology, characterization, mechanical characteristics, and species sources of decellularized corneal-based 3D scaffolds will all be evaluated in this review.

## 2 Source species

Porcine corneas, often referred to as acellular porcine corneas (APCs), are used in several investigations on decellularizing corneas for tissue engineering since they are more plentiful than human donor corneas and share similarities in size and shape. APCs with and without cells have been compared in numerous studies using animal transplant models. The following cell types are examined in conjunction with the APCs: amniotic epithelial cells, corneal stem cells, corneal epithelial cells, corneal stromal cells, which include fibroblasts and keratocytes, and cells that are directly derived from limbal explants [26].

Human Corneas: To minimize the higher risk of rejection, human donor tissue is always chosen over xenogeneic tissue, even if research employing APCs have shown encouraging outcomes. An appealing material for corneal tissue engineering is provided by decellularizing human donor corneas that are turned down for transplantation because of their poor endothelial cell densities. However, as with all organ transplants, there are still only a certain amount of rejected corneas accessible for this procedure because donor corneas are in short supply. People are starting to look into lenticules derived from Small Incision Lenticule Extraction (SMILE) as a donor tissue source as a possible remedy for this issue. The availability of SMILE generated lenticules, which are fragments of stromal tissue removed during refractive surgery, is growing as the procedure becomes more widespread [27,28]. To treat stromal defects or tissue design corneal transplants, SMILE-derived lenticules have attracted attention as transplant materials, either with or without decellularization. It is suggested that they could be utilized allogeneic to augment the pool of donor corneas for patients with stromal deficiencies in need of transplantation, or autologously when changes in corneal shape occur and the thickness needs to be repaired [28].

The application of lenticules for corneal repair has been the subject of numerous investigations. For instance, autologous lenticule re-implantation in rabbits into the same eye from which it was taken revealed that the cornea remained clear and its thickness was restored 28 days after the procedure [29]. In a second autologous trial, a little lenticule from one rabbit eye was transplanted into the stromal pocket of the other eye. Edema was seen in the transplanted eye right after surgery and persisted until day 10. Nerve fiber regeneration was seen one month after the transplant, the lenticule had integrated and the extracellular matrix had gradually removed by three months, and the stroma looked normal by six months [28]. These findings suggest that autologous repair of minor stromal lesions may be possible, much like autologous limbal explants can be used to treat limbal stem cell deficiency (LSCD) in cases when only one eye is impacted. This technique, like allogenic lenticule implantation, has the drawback of only being able to fix minor flaws; it cannot replace or restore an entire cornea. In order to solve this problem, one study combined several decellularized SMILE-derived lenticules into a bigger substrate for corneal tissue engineering using fibrin glue [28]. The construct was able to restore the ultrastructural organization of the cornea without vascularization, degradation, or rejection when it was implanted into a rabbit anterior lamellar keratoplasty model. It also repaired the anterior portion of the stroma with a re-epithelized surface that remained transparent [28]. Since it is clear from the studies above that what works in these healthy transplant models is not always applicable to more extreme cases, the next step in this approach would be to try this type of tissue engineered cornea in a burn or other injury/disease model with a less stable ocular surface [28].

## 3 Types of corneal decellularization methods

**Fig. 1:**
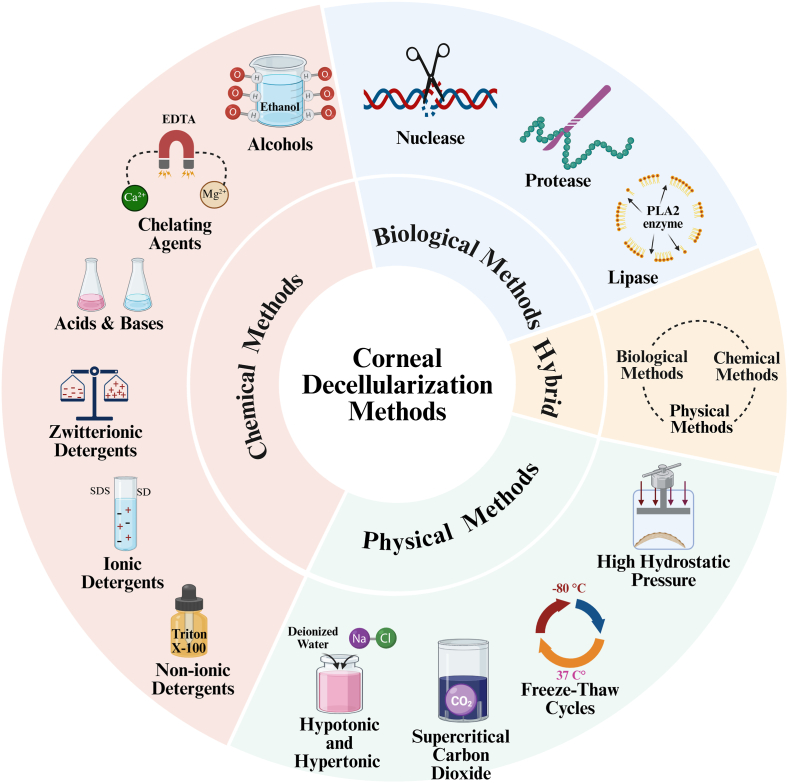
This figure provides a comprehensive overview of diverse methodologies for corneal decellularization, categorized into three primary approaches: chemical, biological, and physical methods.

The ideal decellularization protocol should completely remove cellular material and antigen molecules from the xenograft to diminish a host immune reaction retain structural and functional proteins of the ECM without disruption of the overall tissue matrix, maintain corneal transparency, and support growth of host and corneal cells [30]. It is important to note that every method of decellularization will result in some disruption to the ECM ultrastructure, however optimization of the decellularization process should try to minimize these undesirable effects [31] Fig. 1.

### 3.1 Physical decellularization methods

Freeze-thaw cycles, hypotonic and hypertonic solutions, ultra-high hydrostatic pressure (UHHP), high hydrostatic pressure (HHP), and supercritical carbon dioxide (scCO2) are physical techniques used to decellularize corneas [32].

#### 3.1.1 Freeze-thaw cycles

Repeated cycles of freezing and thawing can be utilized to lyse cells while maintaining the ECM's structure with little disruption and favorable optical characteristics. For corneal decellularization, five to six freeze-thaw cycles are often utilized [31,33]. Porcine corneas were decellularized using freeze-thaw cycles, Triton X-100, and SDS. Although all of the hydrogels made from the ECM of decellularized corneas were extremely transparent, the most transparent ones were those that were decellularized using freeze-thaw cycles. Following decellularization, hematoxylin and eosin (H&E) staining verified the absence of cell nuclei [33,34]. But since this decellularization process cannot effectively remove cells and genetic material by itself, it works best when combined with additional methods (such as chemical detergents or nucleases) [31,33,35,36].

#### 3.1.2 Hypotonic and hypertonic solutions

Hypotonic and hypertonic solutions effectively lyse cells and help rinse cellular remains but not sufficiently enough. To achieve better decellularization outcomes, they can be used in combination with nucleases (such as DNAse and RNAse) which effectively remove nucleotides after cell lysis [31,37]. Though not enough, hypotonic, and hypertonic solutions efficiently lyse cells and aid in rinsing cellular remnants. They can be employed in conjunction with nucleases (such DNAse and RNAse), which efficiently remove nucleotides following cell lysis, to improve decellularization results [[38], [39], [40]]. When 0.2 % Triton X-100 was used to wash corneas pretreated with NaCl, a very effective decellularization result was obtained [41,42]. Triton X-100, poly (ethylene glycol) (PEG), and liquid nitrogen were insufficient to completely remove the cellular material when employing various decellularization techniques to decellularize human corneas. Whole human corneas were also effectively decellularized using NaCl and nucleases while maintaining the ECM's structure [43]. Histological staining and DNA quantification demonstrated that short incision lenticule extraction (SMILE) lenticules were effectively decellularized using 1.5 M NaCl and DNase. Nonetheless, there was some evidence of ECM structural disturbance [44].

#### 3.1.3 High and ultra-high hydrostatic pressure

This technique breaks down cell membranes by applying pressures higher than 600 MPa [34,45]. Porcine corneas were successfully decellularized using HHP and UHHP, removing all cells while preserving the ECM structure. Furthermore, following transplantation into rabbit corneas, the generated scaffolds were shown to be noncytotoxic [46,47]. The potential for single application is this method's biggest benefit [34]. But because of the costly equipment needed, it hasn't been employed often [5,30]. HHP decellularization uses 600–800 MPa for 10–30 min to disrupt cell membranes while maintaining ECM integrity [48,49]. Freeze–thaw methods employ 3–6 cycles between −80 °C and 37 °C to lyse cells [49].

#### 3.1.4 Supercritical carbon dioxide) ScCO2(

ScCO2 is being employed as a substitute decellularization method. ScCO2 keeps the extracellular structure of corneas like natives while drastically cutting down on the decellularization time. By obtaining acellular scaffolds that are compatible with the immune system, it can be employed concurrently to sterilize tissue. This method's drawback is the intricate ScCO2 reactor apparatus required [42,50,51]. When it came to maintaining the ultrastructure, the control group decellularized with 1 % SDS performed worse than the ScCO2 decellularization procedure. ScCO2 has been used to effectively remove cells from cow corneas, as demonstrated by H&E staining [50]. Acellular porcine corneas were also effectively created using ScCO2. DNA quantification and histology staining have verified a full decellularization. Rabbits receiving treated tissue had good biocompatibility [42,52].

### 3.2 Chemical decellularization methods

#### 3.2.1 Detergents

The use of detergents, including ionic, non-ionic, and zwitterionic detergents, is by far the most used technique for decellularization. These successfully break down the cell membrane and separate DNA from proteins [53,54], but they do so at the expense of damaging and eliminating important extracellular matrix proteins [[55], [56], [57]]. Since non-ionic detergents focus on lipid-lipid and lipid-protein interactions rather than protein-protein interactions, they are kinder than ionic treatments [30].

##### 3.2.1.1 Non-ionic detergents

###### 3.2.1.1.1 Triton X-100

A nonionic chemical detergent called Triton X-100 (0.1–2 % v/v, 24–72 h*)* is used to decellularize corneas. Although it is less disruptive than ionic detergents, it is less successful at eliminating cellular structures. [33,38]. When Triton X-100 was used to decellularize corneas at concentrations of 0.1–1 %, there were also reports of an inadequate cell removal [37]. Porcine corneas were sufficiently decellularized with the use of 1 % Triton X-100 and lyophilization (a freeze-drying procedure). After three months of implantation into the rabbit corneal stroma, the acellular matrix's biocompatibility was demonstrated without an immunological response [58]. Porcine corneas were effectively decellularized using a 0.2 % Triton X-100 solution and a hypertonic solution (NaCl) [41,42]. Based on the measurement of remaining DNA, the combination of Triton and NaCl has proven to be even more effective than the supercritical carbon dioxide (ScCO2) and NaCl method in decellularizing pig corneas. Furthermore, compared to corneal scaffolds treated with ScCO2, those treated with Triton exhibited greater flexibility and strength [42]. As demonstrated by H&E and DAPI staining, 2 % Triton X-100 combined with ammonium hydroxide or nucleases has also effectively decellularized ocular tissue [59,60].

##### 3.2.1.2 Ionic detergents

###### 3.2.1.2.1 Sodium Dodecyl sulfate (SDS)

Because of its exceptional ability to remove cells and dissolve cellular membranes, SDS (0.1–1 % w/v, 12–48 h) is the most utilized chemical in corneal decellularization procedures. At varying dosages and exposure durations, research has documented either “complete” or “sufficient” decellularization [32]. Nevertheless, even with the same or longer time-points and greater concentrations, Sasaki et al. failed to reproduce the results [61]. Various ideal circumstances have been proposed by studies. Du and Wu suggested 0.5 % (wt/vol) SDS for 24 h at 4 °C with protease inhibition and continuous shaking (32) and Zhou et al. suggested 0.1 % (wt/vol) SDS for 7 h at 37 °C [62], Gonzalez-Andrades et al. suggested 0.1 % (vol/vol) SDS for 48 h at room temperature with 300 rpm continuous shaking, and so on [32].

###### 3.2.1.2.2 Sodium deoxycholate (SD)

Another ionic detergent for decellularization is SD. At the same dose and incubation period, it is less effective than SDS; also, it disrupts the structure of the extracellular matrix more [63]. After 48 h of incubation, 0.5 % and 1 % SD were able to eliminate all the cornea's cellular structures, but they also destroyed the tissue histoarchitecture, particularly the collagen fibrils and Bowman's and Descemet's membrane. The nuclei and structures of corneal cells were not completely removed when the incubation period was shortened to 24 or 36 h [63]. Cellular material in the decellularized human cornea was not entirely removed by using 0.5 % SD with deoxyribonuclease (DNAse). Higher SD concentrations, however, such as 1 % SD in addition to DNAse, effectively decellularized human corneas in less than a day. Using DAPI labeling and H&E, no stromal or epithelial cells were seen [64].

##### 3.2.1.3 Zwitterionic detergents

3-[(3-Chola-Midopropyl) Dimethylammonio]-1-Propanesulfonate (CHAPS) is a zwitterionic detergent that is insufficiently effective in decellularizing corneas [63]. Cellular structures were not significantly removed by 0.5 % or 1 % CHAPS after 24 h. Even extending the incubation period to 36 h did not result in enough decellularization; instead, it ruined the corneal tissue's histoarchitecture [63]. CHAPS showed the weakest decellularization efficacy on corneas compared to SDS and Triton X-100 with a significant number of nuclei remained [32]. These cellular structure remains could lead to an unfavorable immunological response [30]. To create bioartificial corneas (BACs), decellularized pig corneal stroma was prepared using a mixture of CHAPS, SDS, and a nuclease. Following decellularization, a very low DNA concentration (28.5 ± 5.5 ng/mg) was found; this decellularization was deemed enough. Tissue transparency and biocompatibility were both favorable outcomes of the produced BACs As a result, CHAPS is a useful supplemental decellularization technique, but it is not enough on its own [65].

#### 3.2.2 Acids and bases

There are a few documented methods for decellularizing corneas with acids and bases [41,42,46].

##### 3.2.2.1 Acids

For corneal decellularization, acids are more frequently utilized than bases [66]. Formic acid was shown to be the best organic acid for pig corneal decellularization when compared to acetic acid, citric acid, and formic acid. This is because it preserves tissue transparency and extracellular matrix structure while allowing for the possibility of recellularization. The good decellularization impact of formic acid on pig corneas was further validated by histology research. Acetic acid, however, was insufficient for corneal decellularization [67]. Similarly, the method for decellularizing bovine corneas using ethanol and peracetic acid (at varying doses) was inadequate since there were cell remnants in the periphery of the decellularized tissue [68].

##### 3.2.2.2 Bases

The only method of corneal decellularization that has been reported in the literature is the application of ammonium hydroxide (NH4OH) [66]. 0.1 % ammonium hydroxide (NH4OH) together with 2 % Triton X-100 was used to decellularize the human corneal stroma. Complete decellularization was con2rmed by H&E staining while preserving the structure of the ECM [59,60].

#### 3.2.3 Chelating agent

A chelating substance called ethylenediaminetetraacetic acid (EDTA) (0.1 %) is typically used in conjunction with another decellularization technique (such SDS and trypsin). It has no effect when used alone [32]. Porcine corneas were reported to be decellularized by a protocol that combined 0.1 % EDTA, 0.3 % SDS, hypotonic tris buffer, and aprotinin. Following decellularization, H&E and DAPI staining confirmed a notable but insufficient clearance of cells from the stroma while preserving the ECM structure [69]. After decellularizing bovine corneas with 0.1 % EDTA, aprotinin, and 0.3 % SDS, a remarkably comparable outcome was achieved [70]. Trypsin ethylenediaminetetraacetic acid (TE) solution produced a good decellularization impact on cow corneas. The general structure was preserved, and no cell remnants were found. Furthermore, there was no change in the decellularized tissue's transparency [68]. Human corneal lenticules were fully decellularized using a hypotonic solution of 0.5 % TE while preserving the DNA content and ECM [71].

### 3.3 Biological decellularization methods

Enzymes or serum are examples of biological techniques for corneal decellularization [32].

#### 3.3.1 Enzymes

Although enzymes have shown effective in decellularizing corneas, they cannot eliminate cellular remnants by themselves [32]. Enzymes known as nucleases (DNAse and RNAse) have been effectively employed in corneal decellularization. They made the treated tissue less translucent and drastically decreased the amount of DNA. After pretreatment with a chemical agent or another enzyme, a superior decellularization outcome was obtained [64,72]. DNAse and 1 % SDS were used to decellularize the human corneas that were unsuitable for keratoplasty. This mixture successfully eliminated all cellular components while maintaining the collagen fibrils' alignment [73]. Human corneas were also effectively decellularized using 1 % SDS and DNAse [64]. Porcine corneas have been successfully decellularized using a mixture of chemical detergents (0.5 % SDS and 1 % Triton X-100) and nucleases [36,66]. In the decellularization process, nucleases are frequently utilized to eliminate any remaining genetic material [74]. Another enzymatic tool for corneal decellularization is phospholipase A2 (PLA2). In comparison to native pig corneas, PLA2 in conjunction with 0.5 % SD proved effective in decellularizing pig corneas, removing enough genetic material while maintaining 80 % glycosaminoglycan (GAG). The scaffolds created with this technique maintained their mechanical and optical characteristics with little alteration. Twelve months following animal transplantation, biocompatibility with no rejection was assessed (rabbit lamellar keratoplasty) [75]. When combined with EDTA, the enzyme trypsin effectively decellularizes the cornea. The corneal tissue was sufficiently decellularized by this solution while maintaining its general structure, as well as its high transparency and biocompatibility. In terms of corneal decellularization, the 0.5 % TE solution was even more effective than the 0.5 % Triton X-100 procedure [71].

#### 3.3.2 Serum

Another effective technique for corneal decellularization is the use of serum. SMILE lenticules have been effectively decellularized by bovine serum after 72 h, as demonstrated by histological staining and DNA quantification. There has been no change in the transparency or collagen fibril organization. SMILE lenticules were more effectively decellularized by bovine serum over 72 h than by a mixture of DNAse and NaCl [44]. Electrophoresis and human serum have been effectively employed to decellularize corneal stromal tissue and produce a good biocompatible scaffold. This technique produced decellularized corneal tissue that exhibited optical, biomechanical, and ultrastructural resemblances to native corneas. There was no evidence of rejection following lamellar keratoplasty (LKP) in in vivo experiments. However, electrophoresis was required since treatment with human serum alone would not have adequately eliminated the cellular structures [5,76]. The benefit of this approach is that it eliminates the need for hazardous detergents and extra enzymes. Additionally, it has been demonstrated that transplanted corneas have good biocompatibility, no immunogenicity, and good transparency [77] (Table 1). Typical total decellularization time varies from 24 h (rapid surfactant + DNase methods) [64] to 5–8 days for SDS/NaCl–based protocols [78]. All protocols incorporated multiple phosphate-buffered saline (PBS) or deionized water washing steps (typically 3 × 24 h) to completely remove residual detergents, DNA, and cellular debris [78].

**Table 1:** The summary of corneal decellularization methods discussed in this review.

Decellularization method	Species	Mechanism of action	Advantages/disadvantages	Ref
Physical (Freeze-thaw cycles)	Porcine cornea	Ice crystal formation causes cell lysis.	(i) Good corneal decellularization effect by lysing cells (ii) Need to be used in combination with other decellularization techniques to remove cells (iii) Disruption to ECM structure (iv) Better optical properties of decellularized corneal tissue compared to the use of chemical detergents	[33]
	Porcine cornea			[35]
Physical (Hypertonic and hypotonic solutions (mostly NaCl))	Human cavader cornea	Detaches DNA from proteins.	(i) Efficient decellularization achieved when in combination with other decellularization method (for example, with nuclease or Triton X-100) (ii) Minimal disruption in the ECM structure (iii) Good optical properties (iv) Less sufficient compared to chemical detergents	[38]
	Human cornea stromal created by femtosecond laser			[38]
	Amniotic epithelial cell and acellular porcine cornea			[41]
	Porcine cornea			[42]
	SMILE			[44]
	Discarded human cornea			[60]
	Human cornea unsuitable for transplantation			[72]
Physical (HHP, UHHP))	Porcine cornea	Increase in pressure results in cell lysis.	(i) Efficient decellularization (ii) Minimal changes in the ECM structure (iii) Solitary application (iv) Expensive equipment required	[46]
	Porcine cornea			[47]
Physical (ScCO2)	Porcine cornea	It bursts cells with high fluid pressure and rapid depressurisation.	(i) Effective corneal decellularization (ii) Reduces decellularization time (iii) Can cause changes in ECM structure (increase gap between collagens 2brils) (iv) Can be simultaneously used to sterilize corneal tissue (v) Less expensive compared to HHP (vi) Limitation is the need of the complex scCO2 reactor system	[42]
	Bovine corneas			[50]
	Porcine cornea			[52]
Chemical (Triton X-100)	Porcine cornea	Breaks up lipid-lipid and lipid-protein interactions.	(i) Not all studies documented sufficient decellularization of corneal tissue (ii) For an efficient corneal decellularization, must be used in combination with other decellularization methods (iii) Less effective than ionic detergents (SDS and SD) but also less disruptive (iv) Can cause minimal changes in ECM structure	[36]
	Human cavader cornea			[37]
	Amniotic epithelial cell and acellular porcine cornea			[41]
	Porcine cornea			[42]
	Discarded cornea of eye donors			[59]
	Discarded human cornea			[60]
	Human cornea unsuitable for transplantation			[72]
Chemical (SDS)	Porcine cornea	Solubilizes cell membranes and dissociate DNA from protein. Disrupts protein-protein interactions.	(i) Very efficient in removing cellular structures and genetic material (ii) Can induce immunologic reaction; must be fully washed after decellularization process (iii) Can cause changes in the ECM structure (iv) Transparency can be lower at higher concentration	[63]
	Human cornea unsuitable for transplantation			[72]
	Human & porcine stromal lenticules			[79]
	Decellularized squid mantle scaffold (DSMS)			[80]
Chemical (SD)	Porcine cornea	Solubilizes cell membranes and dissociates DNA from protein. Disrupts protein-protein interactions.	(i) Effective decellularization method (ii) Less effective than SDS (often used with other methods, mostly nucleases) (iii) Disruptive for collagen 2brils (less than SDS)	[63]
	Human cornea			
Chemical (CHAPS)	Porcine cornea	Has properties of non-ionic and ionic detergents.	(i) Ineffective for corneal decellularization when used alone (ii) Effective when used with another decellularization method (such as SDS) (iii) Destruction of histoarchitecture of corneal tissue at longer incubation time	[63]
	Bioartificial cornea			[65]
Chemical (Acids)		Solubilizes the cytoplasmic components of cells and the removal of nucleic acids by causing or catalyzing hydrolytic degradation of biomolecules	(i) Not all studies document sufficient corneal decellularization (ii) Slight changes in ECM structure	[81]
	Discarded human cornea			[60]
	Porcine cornea			[67]
	Bovine cornea			[81]
Chemical (Bases)	Discarded cornea of eye donors		(i) Only the use of ammonium hydroxide (NH4OH) has been documented for corneal decellularization in the literature (ii) Need to be used with another effective decellularization method	[59]
	Discarded human cornea			[60]
Chemical (EDTA)	Porcine cornea	Dissociates cells by separating metal ions.	(i) Incomplete cell removal when used alone (ii) Efficient decellularization when combined with another decellularization method (for example, SDS and trypsin)	[81]
	Bovine cornea			[70]
	Human cornea			[71]
	Human cornea			[73]
	Bovine cornea			[81]
Biological (Nucleases (RNase and DNase))	Porcine cornea	Cleaves nucleic acids and aid in their removal.	(i) Often used in decellularization protocols to help remove residual genetic material after cell lysis (ii) Must be used with another decellularization method (iii) Can disrupt ECM structure (iv) Less transparent decellularized corneal tissue	[82]
	Porcine cornea			[82]
	Porcine cornea			[83]
Biological (PLA2)	Porcine cornea	Hydrolyzes phospholipid components of cells.		[75]
	Cat cornea			[84]
	Acellular porcine limbal stroma			[85]
	Porcine cornea			[86]
Biological (Trypsin)	Porcine cornea	Hydrolyse Protein and disrupts protein-protein interactions.		[82]
	Porcine cornea			[82]
Biological (Serum)	SMILE	Serum nucleases degrade DNA and RNA.	(i) Efficient decellularization method (ii) Maintained ultrastructure of decellularized corneal tissue (iii) Good optical properties after decellularization (iv) No need for other decellularization methods	[44]
	Porcine cornea			[76]
	Porcine cornea			[77]

## 4 Corneal characterization methods

**Fig. 2:**
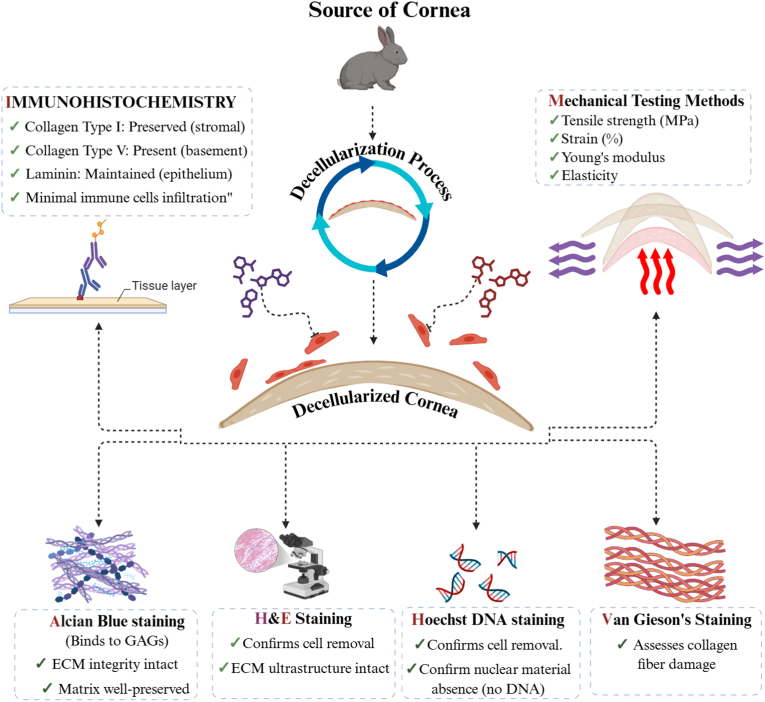
Schematic illustration of corneal tissue undergoes decellularization to produce acellular corneal scaffolds, which are subsequently characterized through immunohistochemistry, mechanical testing, and histological staining methods to confirm extracellular matrix preservation, cellular removal, and structural integrity for tissue engineering applications.

The effectiveness of corneal decellularization and the biocompatibility characteristics of the resulting scaffold have been assessed using a variety of techniques. Histological staining is frequently the initial test used to assess if cellular debris has been removed and whether the corneal ECM ultrastructure has been maintained [30]. The general histoarchitecture of the treated cornea and the extent of decellularization are frequently assessed using H&E staining. A cationic dye called Alcian Blue staining, which binds to GAGs [87], is used to show how much the ECM has been disrupted. Van Gieson's staining has been employed to evaluate the degree of collagen fiber damage [55]. DAPI or Hoechst DNA staining [6,55], which are fluorescent molecules that attach to AT clusters in DNA, can be used to confirm the lack of nuclear material [23]. Certain proteins, such as collagens type I and V [9,37,55,88] and laminin, which are found in the cornea's epithelial layer, have been found using immunohistochemistry. The immunogenicity of the acellular pig cornea has also been assessed by immunostaining [88]. The Galα1-3Galβ1-4GlcNAc-R (αGal) [9,39] epitope has been detected by binding experiments; αGal can cause hyperacute rejection of a xenotransplant [88]. Transparency and refractive index critically determine the optical performance of decellularized corneal scaffolds. The transparency of decellularized porcine corneas typically ranges from 85 % to 95 % transmittance across the visible spectrum (380–770 nm), comparable to that of native human corneas (87–92 %) [89,90]. In freshly prepared transparent decellularized corneas, the refractive index (1.367 ± 0.001) remains only slightly lower than natural cornea values (1.373–1.380) [90,91]. Preservation of uniform collagen fibril spacing and minimal stromal swelling are essential for maintaining refractive homogeneity and light transmittance [91] Fig. 2.

## 5 Mechanical properties

Decellularized corneal scaffolds have shown excellent mechanical strength, most likely as a result of the ECM structure being preserved. [28]. Evaluation of the biomechanical and optical characteristics of the acellular scaffold is crucial because corneal strength depends on the preservation of an ordered stromal extracellular matrix, which might be disturbed during decellularization. Given that the cornea is a load-bearing tissue, its mechanical and viscoelastic properties are crucial to preserving its functionality. In order to ascertain if decellularization resulted in a decrease in strength or elasticity, the mechanical characteristics of decellularized corneas have been investigated using extensiometry or inflation tests [30]. The matrix structure would be preserved if these characteristics were maintained [81].

By measuring the integration of the scaffold and the host immune response after implantation, the biocompatibility of decellularized corneas has been assessed in vivo. For this, pig-to-rabbit models are typically employed. An acellular cornea or cell-seeded decellularized corneal construct is sutured into a stromal pocket that has been created, with native corneas serving as a control. By analyzing the cornea's opacity and neovascularization at specific time points after implantation, the transplant's success can be evaluated macroscopically [30]. The integration of the scaffold with the host has been observed by cells infiltrating the transplanted cornea, and H&E staining can validate the infiltration of neutrophils and macrophages in and around the donor tissue [6,30,69,92]. Table 2 is a quantitative summary detailing tensile and elastic characteristics from published datasets.

**Table 2:** Mechanical properties of the cornea in different decellularization methods.

Sample	Tensile Strength (MPa)	Young's Modulus (MPa)	Ref
Native cornea	3.5–5.0	0.3–0.6	[89]
SDS 0.5 % treated	3.0–4.2	0.25–0.5	[49]
SLG + supernuclease	10.6 ± 0.5	–	[89]
HHP-treated	3.8–4.6	0.28–0.55	[48]

## 6 Corneal recellularization

**Fig. 3:**
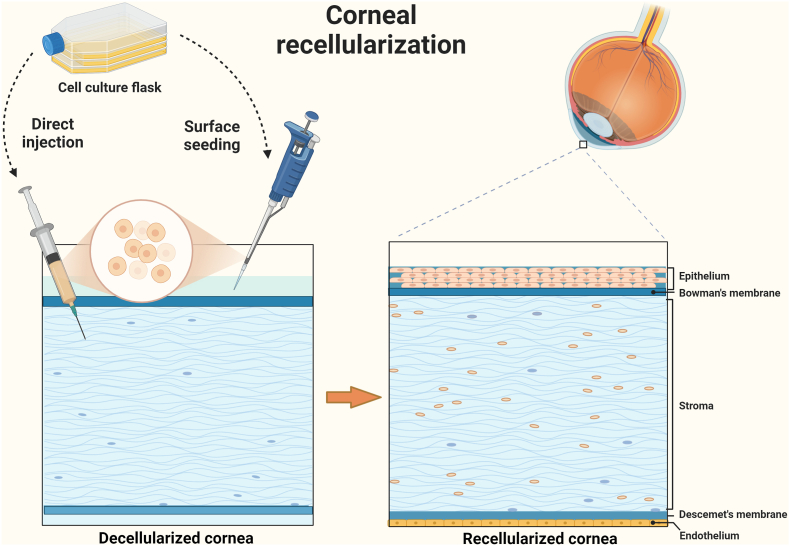
Corneal recellularization.

Decellularized corneas must be able to sustain corneal cell development in order to be utilized as scaffolds [37,39]. In order to restore a corneal stroma, epithelium, or endothelium, cells can repopulate onto decellularized corneal scaffolds, which offer a matrix akin to the natural cornea [37,39]. Porcine, bovine, and feline corneas have been decellularized and reseeded with various corneal cell types for use in animal models [30,55]. When repopulating a decellularized cornea, the seeding density is crucial because it not only ensures a confluent layer of cells on and across the scaffold, but also prevents contact inhibition [30]. Standard histological/immunohistological techniques, in addition to SEM, TEM, and optical studies, as well as cell proliferation and cytotoxicity assays, can all be used to assess the success of recellularization [30] (Fig. 3). In rabbit models, anterior lamellar grafts are typically 6–8 mm in diameter and 100–200 μm thick [93]. Porcine corneal models employ full-thickness 1 × 2 cm discs for large-area transplantation [90]. The rabbit eye (∼16–19 mm axial length) and pig eye (∼23.9 mm) are proportionally comparable to the human cornea (∼24 mm), supporting their translational relevance [94].

### 6.1 Stromal recellularization

To recellularize the corneal stroma, there are two primary methods. In the first, cells are seeded onto the scaffold's surface. It has been demonstrated that human keratocytes sown on the surface of an acellular cornea move, disseminate, and develop inside the scaffold [39]. After 21 days in culture, bovine keratocytes sown onto decellularized corneal buttons showed a confluent layer of cells creating networks of interconnected cellular processes [81]. Injecting cells straight into the scaffold is an alternate method of seeding cells on top of it. By injecting the cells into the cornea, isolated human corneal stromal cells effectively recellularized scaffolds treated with SDS [95]. In addition to positive staining for lumican, keratocan, and collagen type 1, the presence of nuclei in the stroma verified the reconstruction of the stroma. Additionally, the combination of injecting cells into a decellularized scaffold and seeding cells on top has been investigated [92]. On an acellular porcine matrix, a corneal anterior lamellar was created using rabbit keratocytes and epithelial cells. A scaffold that resembles natural rabbit corneas was created by planting rabbit corneal epithelial cells on its surface and injecting keratocytes into the scaffold's matrix. The expression of vimentin in the stromal layer and cytokeratin 3 in the epithelial layer was validated by immunohistochemical studies [30].

### 6.2 Epithelial recellularization

The disease known as limbal stem cell deficit, in which the stem cells that are responsible for renewing the corneal epithelium have been lost, has been examined in relation to corneal epithelium reconstruction [37]. It has been observed that human corneal epithelial cells plated on the basement membrane easily adhere to the cornea and express keratin 12. Proliferation was visible through Ki67 staining. Aldehyde dehydrogenase 1 (ALDH1), a hallmark of corneal keratocytes, was expressed by fibroblasts that were injected into the stroma to aid in the establishment of the epithelial layer. These cells had a native and spread shape. By allowing the establishment of two cell layers on the matrix's surface, rabbit corneal limbal epithelial cells seeded onto the surface of a pig cornea decellularized with 0.5 % SDS validated this acellular matrix as an appropriate substrate for the production of epithelial layers [55].

### 6.3 Endothelial recellularization

In order to address hereditary conditions like Fuch's Dystrophy that can result in corneal opacification, decellularized human corneas have been effectively seeded with human corneal endothelial cells [59]. Because the endothelium uses Na+/K + -ATPase pumps to keep the stroma dehydrated, it is vital to human eyesight [96]. Direct seeding of human corneal endothelial cells onto a decellularized stroma results in a monolayer of cells that retains the expression of functional and junction biomarkers, including as ZO-1, connexion 43, and Na+/K + -ATPase [59]. A clonal endothelial cell line, B4G12, has also been used to create human corneal endothelium equivalents on acellular pig corneas [97]. The denuded endothelium of each scaffold was replaced by a monolayer of cells that stained positively for the markers ZO-1, which showed that the cells had formed a closed monolayer, and Na+/K + -ATPase, a protein complex that is involved in the function of the corneal pump. On the rear surface of the corneas of cows, human endothelia developed into a continuous, densely packed monolayer [70]. The rebuilt endothelium layer exhibited normal intercellular barriers, intracellular connections, and pump functions, as demonstrated by immunohistochemical labeling of cytokeratin 3, ZO-1, connexin 43, Na+/K + -ATPase, and Na+/HCO3- [30].

## 7 Application of decellularized cornea in composites

Using composites offers the opportunity to gather admired characteristics of several materials together in one product. Three-dimensional (3D) bioprinting technology is an outstanding tool to construct favorable composites with desired materials [98]. This technology has revolutionized the field of regenerative tissue engineering makes it feasible to create personalized corneas. Research by Zhang et al. showed the effectiveness of a single bionic for 3D bioprinter, which was the composition of porcine decellularized corneal ECM/gelatin methacryloyl. In vivo results showed the hydrogel offered high transparency and excellent biocompatibility; it also stimulated epithelialization and restored clarity [99].

Another group cross-linked porcine decellularized corneal stroma matrix (pDCSM) with methacrylated hyaluronic acid (HAMA) to develop a hybrid hydrogel. Because of the non-competitive dual-crosslinking process mechanism, the adhesive properties of the obtained material were high to achieve suture-free repair, and had equal transparency with the cornea, while mechanical characteristics allowed it to work under increased intraocular pressure. When used in a rabbit model, it adhered intimately to the stroma bed with long-term retention. This hybrid hydrogel promoted corneal re-epithelialization and wound healing after being implanted in the corneal tissue [100].

Even though decellularized corneal matrix-based bioprinter construct have been observed to possess promising biological functionality, researchers have pointed out that the acellular scaffolds have a drawback of being mechanically stiff. To address this, one team blended the matrix with another natural polymer: sonicated silk fibroin. They successfully 3D printed the anisotropic corneal stroma equivalent with regionally distinct properties by positioning the mechanically robust outer rim and the more transparent central zone with only decellularized corneal matrix (DCM). This hybrid bioink displayed a high complex modulus over that of the pure DCM while sustaining structural integrity without compromising optical clarity due to the presence of crystalline beta-sheet conformation within the hydrogel [101].

**Fig. 4:**
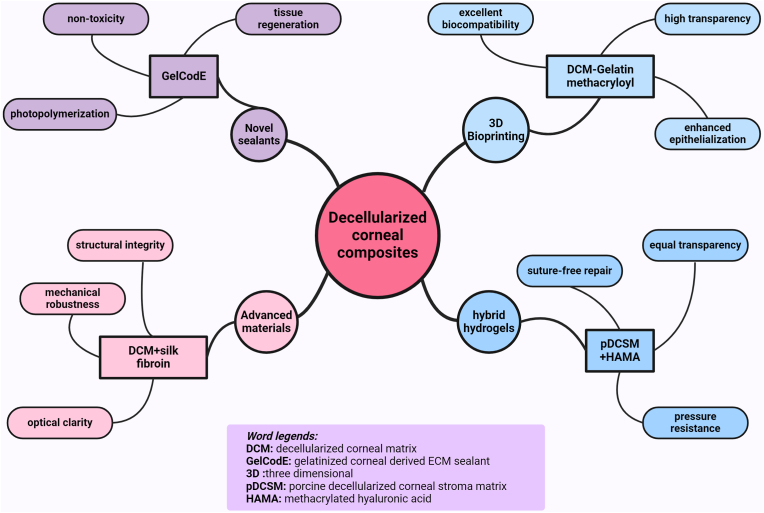
Key properties and components of advanced decellularized corneal matrix (DCM)-based composites for corneal repair.

Based on these inventions, a new gelatinized corneal derived porcine ECM sealant called GelCodE was created. It relies on the matrisome in charge of tissue-organization and development within GelCodE while visible light-based photopolymerization with ruthenium/sodium persulfate quickly form a covalent bond with the adjacent tissue. The factors including ease of application, non-toxicity and tissue regeneration capabilities make GelCodE to be a suitable candidate for use in clinical applications. Interestingly, the regenerated tissues are shown to have normal matrix properties with little scar tissue formation [102]. Altogether, these works illustrate that decellularized corneal matrices are a promising substrate for generating biomimetic composites for the repair of damaged corneas. Utilizing the native architecture of the corneal tissue as a guide, scientists have created hydrogels, hybrid materials, as well as printed constructs that mimic the structure, mechanical and optical characteristics of the natural tissue (Fig. 4).

## 8 Application of decellularized cornea

Recently, decellularized corneas have been studied for tissue engineering as scaffolds. Using decellularized cornea in tissue engineering is a promising way for treating corneal blindness as well as ocular surface disorders. The unique properties both structural and biochemical of decellularized corneal scaffolds make them competent for tissue engineering applications in corneal region. Initial source was cadaveric donor corneas. They were not good choices for transplantation, since positive serology or nonviable epithelium/endothelium and low cell counts [78,[103], [104], [105]]. Therefore, researchers concentrated on cornea decellularization. Focusing on entire cornea decellularization besides thin slices of 90–200 μm corneal stroma using microtome or cryotome and femtosecond laser to obtain corneal lamellae have been reached to valuable findings in the literature [59,106,107].

We mentioned previously several corneal decellularization methods have been ran to reach to a proper balance of saving the biomechanical structure and optical function of the corneal tissue and removing cells to prevent immune rejection. There are also studies which tried to validate the final product in vivo. The safety and biocompatibility of decellularized cornea were tested successfully in several studies with no short- or long- term immune reactions by embedding them into rabbit corneal stroma [106,108,109]. Small incision lenticule extraction (SMILE) is a procedure for correcting myopia that its byproduct named lenticule is being used for corneal tissue engineering. Yam et al. decellularized extracted lenticules using 0.1 % SDS, produced a transplantable scaffold with native-like stromal structure and components and successfully transplanted that to a rabbit stromal pocket [108].

**Fig. 5:**
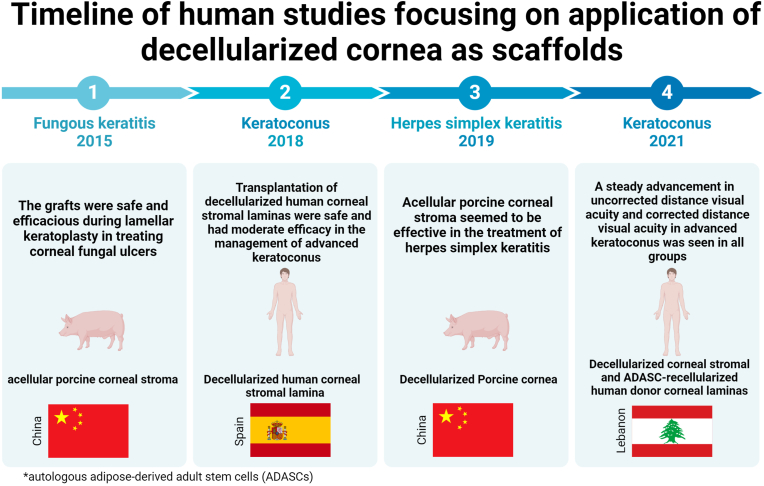
Timeline of translational research reporting the efficacy and safety of decellularized corneal scaffolds (human and porcine) used in the management of diverse corneal disorders, including herpes simplex keratitis, keratoconus, and fungal keratitis, in overseas clinical trials.

In 2015, a bioengineered corneal product developed by CRMI a Chinese corporation won approval from regulators, China's FDA. The procedure includes treating pig corneas using sodium chloride and Triton X-100, followed by glycerol treatment and cobalt-60 sterilization [110]. Originally, 47 patients suffering from fungous keratitis were treated during the first phase of clinical testing. In a subsequent study, the focus was on its efficiency in the treatment of herpes simplex keratitis where patients' stress was caused by inadequacy of engineered tissue substituting human transplants. However, human grafts were not always successful, and some patients had to be transplanted with traditional humanized donor tissues when the engineered tissue started to degrade. Rapid improvements were seen in patients' inflammatory conditions, neovascularization, and transparency of the cornea although the first grafts were clouded. But in more than 1000 scaffolds implantations, analysis of the long-term effect of more than five years on those 10–13 patients has not been reported [111]. The doubts remain, however, if such method was suitable for the treatment of other corneal diseases except fungal. There are also two clinical trials. One which ran in Spain with completed phase 1, analyzed the safety and effectiveness of the transplantation of the decellularized human corneal stromal lamina with or without the recellularization of autologous adipose-derived adult stem cells (ADASCs) in the corneal stroma in the patients with advanced keratoconus. It was concluded from their finding that transplantation of decellularized human corneal stromal laminas is safe and has moderate efficacy in the management of advanced keratoconus. Whether this can have positive impact from recellularization using autologous adipose-derived adult stem cells remains uncertain [112]. Another study revealed corneal stromal cell therapy with three treatment groups: the intrastromal implantation with ADASCs, and decellularized or ADASC-recellularized human donor corneal laminas in advanced keratoconus. All groups didn't show any complications during three years after treatment and interestingly, a steady advancement in uncorrected distance visual acuity and corrected distance visual acuity in advanced keratoconus was seen in all groups, especially in decellularized or ADASC-recellularized human donor corneal laminas groups [113] (Fig. 5, Table 3).

**Table 3:** Summary of human studies focusing on application of decellularized cornea as scaffolds for tissue engineering.

Country	Source for scaffold	Summary of findings	Targeted disease	Ref
China	acellular porcine corneal stroma	The grafts are safe and efficacious during lamellar keratoplasty in treating corneal fungal ulcers	fungous keratitis	[110]
China	Decellularized Porcine cornea	Acellular porcine corneal stroma seems to be effective in the treatment of herpes simplex keratitis	herpes simplex keratitis	[111]
Spain	decellularized human corneal stromal lamina	transplantation of decellularized human corneal stromal laminas is safe and has moderate efficacy in the management of advanced keratoconus.	keratoconus	[112]
Lebanon	Decellularized corneal stromal and ADASC-recellularized human donor corneal laminas	a steady advancement in uncorrected distance visual acuity and corrected distance visual acuity in advanced keratoconus was seen in all groups	keratoconus	[113]

## 9 Challenges and limitations

There is still a long way in development of bioengineered cornea and requires substantial research to be considered the best choice in corneal transplantation. The laboratory processes are limited for removing cells from the initial tissue and are useful for small scales. To bring this technology to bedside, scaling up these procedures besides preserving sterile conditions is essential. Additionally, the scientific community must establish standardized criteria for evaluating scaffold quality, particularly regarding the elimination of donor cells and maintenance of the ECM structure [114]. The lack of studies that focused on determining the most proper cell types for recellularization and developing effective methods for reintroducing cells to the scaffolds is obvious. There is a need for development of ready-to-use scaffolds for the conditions that recellularization is not necessary. Finally, this new approach needs to prove its advantages as a suitable alternative to traditional corneal transplants to become the top choice, and this is a process that requires a lot of research [66].

## 10 Benefits and prospects

It has been claimed that about 10 million people worldwide have bilateral corneal blindness [115]. Corneal transplant, or keratoplasty is the only way to treat severe cases. Despite avascular nature of cornea as being an immune privileged organ, its transplants can face graft rejection, which is the most common cause of transplant failure for corneas [116]. On the other hand, the quality of the cadaveric tissues for transplantation according to the age of the deceased person and the time since his/her death, reduces the success rate of this treatment method [117].

The decellularized tissue grafts might be prepared for partial-thickness transplant procedures for those patients in whom the corneal stroma is clouded but the endothelial layers are healthy. This would also increase graft availability and enable healthcare systems to deal more effectively with corneal blindness. The transparency is a critical characteristic of a grafted decellularized cornea. High proportion of studies show acceptable level of transparency of grafted corneal composites immediately or a few weeks after transplantation (10.1039/D5TB00090D, https://doi.org/10.1016/j.ijpharm.2024.124510), which is initially attributed to maintaining the original extracellular matrix structure and the gradual repopulation by host keratocytes, facilitating the restoration of stromal organization and optical clarity. The strategy of rendering unsuitable donor tissue into workable grafts is a very promising solution to the shortage of cadaver corneas, despite several obstacles before this approach can realize clinical translation. Development here, however, requires establishment of standards for cell removal that balance immune compatibility with tissue function, coupled with robust testing protocols [78].

## 11 Conclusion

Decellularized corneal scaffolds can further both the science of corneal transplantation and tissue engineering. Widespread applications of decellularized cornea and its combination in the composites highlights the potential of decellularized corneal scaffolds as an innovative solution for corneal transplantation. Whereas there has been significant advancement within this modality of therapy, further scientific inquiry is needed to optimize their clinical utility. Preserving some key elements like corneal transparency during decellularization and recellularization of the cornea is essential to develop a functional and effective scaffold. Future developments are likely to be based on novel technologies, combined with an improved understanding of corneal regeneration and biological processes. Establishing such a unified approach and optimizing its in vivo biocompatibility would be crucial for advancing corneal tissue engineering and transplantation technologies.

## Funding

Not applicable.

## Declaration of competing interest

The authors declare no conflicts of interest with respect to the research, authorship, and/or publication of this article.
